# Supplementary material for: Analyzing the performance of metaheuristic algorithms in speed control of brushless DC motor: Implementation and statistical comparison
Source: PLoS One. 2024 Oct 31;19(10):e0310080. doi: 10.1371/journal.pone.0310080 (PMC11527228; doi:10.1371/journal.pone.0310080)
Supplement: Attachment — Submitted filename: CommentsToAuthors.pdf [file pone.0310080.s002.pdf]

Comments to authors

This paper presents the implementation and statistical analysis of metaheuristic algorithms in speed control of brushless DC motor. However, there are some issues that need to be resolved.

(1) The manuscript needs careful editing and particular attention to English grammar, spelling, punctuation marks and sentence structure.

(2) In the introduction, the references provided by the author are not up to date.

(3) There are some typo errors in the manuscript, for instance, in Section 4, ”trajectorybased” should be ”trajectory based”.

(4) It is evident that there are some errors in the formulae, for instance, Eq. (1) and Eq. (2).

(6) The style of the images and tables is not uniform, and the quality is not optimal. It is recommended that the images and tables be modified to enhance their aesthetic appeal.

(7) Some errors have been identified in the data entry process within the tables, for instance, Table 10.

(8) It is imperative that the author elucidate the specific meanings of these indicators in the simulation.
