# Supplementary material for: Analyzing the performance of metaheuristic algorithms in speed control of brushless DC motor: Implementation and statistical comparison
Source: PLoS One. 2024 Oct 31;19(10):e0310080. doi: 10.1371/journal.pone.0310080 (PMC11527228; doi:10.1371/journal.pone.0310080)
Supplement: Attachment — Submitted filename: Sheet-19658.docx [file pone.0310080.s001.docx]

This paper presented the Analyzing the Performance of Metaheuristic Algorithms in Speed Control of Brushless DC Motor: Implementation and Statistical Comparison. The paper is organized, and the authors describe objectives and methodology of their work. Suggest this paper could be reconsidered after revision. The following comments need to be incorporated in this revision.

1. Please ensure all abbreviations are explained the first time they are mentioned.
   Abstract should be improved. Explain a problem and its proposed solution and benefits over existing strategies.
2. How to set optimal parameters in your model? You use standard heuristic model thus optimization of parameters is necessary to make it more efficient.
3. What are the needs and benefits of this paper? What are the challenges and contributions to this field? 4. Why a new method is needed? What are the limitations of existing ones? After a comprehensive discussion about the contributions and novelties, please, list them briefly. Please explain more clearly.
4. The background of the research is rather weak. The authors should provide a more thorough overview of the scientific problem and show unresolved issues.
5. What is the need of a new algorithm, when effective optimization algorithms are available in the literature?
6. Introduction is vaguely written. My suggestion is to divide the introduction into three subsections: 1) motivation and incitement, 2) literature review and 3) contribution and paper organization.
7. Improve the quality of figures.
8. There is no discussion on the cost effectiveness of the methods surveyed. What is the computational complexity? Please include such discussions.
9. There is no statistical test such as CEC 2019, 2017 and CEC 2022, to judge about the significance of the method's results. Without such a statistical test, the conclusion cannot be supported
