# Supplementary material for: Analyzing the performance of metaheuristic algorithms in speed control of brushless DC motor: Implementation and statistical comparison
Source: PLoS One. 2024 Oct 31;19(10):e0310080. doi: 10.1371/journal.pone.0310080 (PMC11527228; doi:10.1371/journal.pone.0310080)
Supplement: Attachment — Submitted filename: Response to Reviewers.docx [file pone.0310080.s003.docx]

**Response to Reviewers**

The authors are thankful to the editor and the reviewers for their valuable time in providing their important and critical comments to improve the manuscript. We have provided the responses below point-by-point for all of the comments.

**Response to Reviewer 1 Comments**

This paper presented the Analyzing the Performance of Metaheuristic Algorithms in Speed Control of Brushless DC Motor: Implementation and Statistical Comparison. The paper is organized, and the authors describe objectives and methodology of their work. Suggest this paper could be reconsidered after revision. The following comments need to be incorporated in this revision.

1. Please ensure all abbreviations are explained the first time they are mentioned.
   Abstract should be improved. Explain a problem and its proposed solution and benefits over existing strategies.

**Response:**

Thank you so much for your valuable feedback. We really appreciate the time you took to review our manuscript and share your constructive comments.

We apologize for not including the full names of all used abbreviations in the manuscript when first time they are mentioned, for example the name of software tools like MATLAB, and SPSS, moreover we have missed some other abbreviations like EMF to write its full form at the first place where it is mentioned. These mistakes are fixed that now by making sure we explain all abbreviations the first time they appear in the manuscript. This will make it easier for readers to understand. For our convenience the following table is mentioned in which we have listed the full form of all used abbreviations. Moreover, these corrections and additions are also done in the actual manuscript.

| **Term** | **Abbreviations** |
| --- | --- |
| Brushless DC Motor | BLDC Motor |
| Proportional-Integral | PI |
| Whale Optimization Algorithm | WOA |
| Particle Swarm Optimization | PSO |
| Ant Colony Optimization | ACO |
| Accelerated Particle Swarm Optimization | APSO |
| Levy Flight Trajectory Based Whale Optimization Algorithm | LFWOA |
| Chaotic Map Incorporated with LFWOA | CMLFWOA |
| Integral Square Error | ISE |
| Statistical Package for Social Sciences | SPSS Software |
| Electromotive Force | EMF |
| Proportional-Integral-Derivative | PID |
| Non-Polynomial | NP |
| Ziegler-Nichols | ZN |
| Modified Ziegler-Nichols | MZN |
| Tyreus-Luyben | TL |
| Integral Time Square Error | ITSE |
| Fractional-Order Proportional Integral Derivative | FOPID |
| Genetic Algorithm | GA |
| Integral Absolute Error | IAE |
| Snake Optimizer | SO |
| Chaotic Online Differential Evolution | CODE |
| Cascaded Short Term Hydro-Thermal Scheduling | CSTHTS |
| Analysis of Variance (Statistical Test) | ANOVA |
| Brushless Permanent Magnet Motor | BPM Motor |
| Levy Flight Distribution | LFD |
| Matrix Laboratory (Software) | MATLAB |
| Honestly Significant Difference | HSD |

The improved version of abstract is also added in below;

A brushless DC (BLDC) motor is likewise called an electrically commutated motor; because of its long help life, high productivity, smaller size, and higher power output, it has numerous modern applications. These motors require precise rotor orientation for longevity, as they utilize a magnet at the shaft end, detected by sensors to maintain speed control for stability. In modern apparatuses, the corresponding, primary, and subsidiary (Proportional-Integral) regulator is broadly utilized in controlling the speed of modern machines; however, an ideal and effective controlling strategy is constantly invited. BLDC motor is a complex system having nonlinearity in its dynamic response which makes primary controllers inefficient. Therefore, this paper implements metaheuristic optimization techniques such as Whale Optimization Algorithm (WOA), Particle Swarm Optimization (PSO), Ant Colony Optimization (ACO), Accelerated Particle Swarm Optimization (APSO), Levy Flight Trajectory-Based Whale Optimization Algorithm (LFWOA); moreover, a chaotic map and weight factor are also being applied to modify LFWOA (i.e., CMLFWOA) for optimizing the PI controller to control the speed of BLDC motor. Model of the brushless DC motor using a sensorless control strategy incorporated metaheuristic algorithms is simulated on MATLAB (Matrix Laboratory)/Simulink. The Integral Square Error (ISE) criteria is used to determine the efficiency of the algorithms-based controller. In the latter part of this article after implementing these mentioned techniques a comparative analysis of their results is presented through statistical tests using SPSS (Statistical Package for Social Sciences) software. The results of statistical and analytical tests show the significant supremacy of WOA on others.

2. How to set optimal parameters in your model? You use standard heuristic model thus optimization of parameters is necessary to make it more efficient.

Response:

Thanks for sharing your thoughts on how we can improve the parameter optimization in our model. We really value your feedback and want to address your concerns with a detailed response.

We understand how crucial it is to optimize the parameters of the metaheuristic algorithms that are used to fine-tune the gains of the controller for motor speed control. It's really important to get these parameters just right! To set these key parameters a combination of the grid search (set boundaries for parameters of algorithms) and random search is utilized. For instance, in the following position update equation of APSO;

$$p_{i}\left(t+1\right)=\left(1-k_{2}\right)p_{i}(t)+k_{2}p_{g\_best}+k_{1}\eta$$

The accelerating coefficients k_1_ and k_2_ should be in the range of 0.1 to 0.4 and 0.1 to 0.7 respectively, in APSO computational code a randomization is involved to set the value of these controlling parameters to any desired value within the range.

In the main manuscript we have mentioned the details and the impact of these optimized parameters on the performance of the controller. We believe that all of these enhancements by considering your comments strengthen our study significantly.

3. What are the needs and benefits of this paper? What are the challenges and contributions to this field? 4. Why a new method is needed? What are the limitations of existing ones? After a comprehensive discussion about the contributions and novelties, please, list them briefly. Please explain more clearly.

Response:

Thank you for this constructive feedback. In view of this, we want to respond back and provide further clarification with respect to the needs, benefits, challenges, and contributions of our paper, as well as the need and benefits for our proposed method over the existing ones. We go through one by one, all your points.

**Needs:**

- Optimal speed control is essential in various industrial appliances.
- Conventional methods are inefficient to provide optimal solutions to complex non-linear systems.
- A controller which can coup with continuously as well as sudden disturbances in the system, is highly demanded in field.

**Benefits:**

- Metaheuristic algorithms provide optimal solutions and precisely control the speed of motor.
- Metaheuristic algorithms provide an efficient approach of finding the best solution as compared to conventional techniques.
- These techniques make the controller more adaptable for varying conditions of the system.

**Challenges:**

- The process of fine-tuning the controller gains for motor speed control can be pretty complicated because there's a huge and intricate search space involved.
- One of the big challenges when it comes to optimizing controller gains for motor speed control is dealing with the computational cost of the optimization process. Therefore, a balance is essential to apply it to real world problems.
- Designing a method adaptable to different types of motors and control systems is one of the challenges in controller gains optimization with regard to motor speed control.

**Contributions:**

- We have tested controller gains optimization with WOA, improved versions of WOA (levy flight trajectory based, and chaotic map incorporated with Levy flight based WOA), PSO, APSO, and ACO algorithms for speed control of a motor.
- We have included in our paper a comparison of algorithms that offers insights into each algorithm's execution and suitability in various scenarios (in terms of number of search particles and type of algorithms).
- We have performed statistical analysis in SPSS software to validate the significance of our results in ensuring that these findings are robust.
- This constitutes, anyway, a good groundwork for possible research in methods of advanced optimization applied to control systems in the future.

**Need for a new method:**

Literature is available with a lot of metaheuristic algorithms for the tuning of controller gains; however, new methods are highly required, particularly in the speed control of the Brushless DC (BLDC) motor using a Proportional-Integral controller. The major problem with most algorithms in the literature is that they tend to run with limitations once applied to special characteristics of the BLDC motor, which must be exactly tailored in order to handle its high efficiency and fast dynamic response. Traditional approaches may not utilize the special dynamics and control requirements of a BLDC motor and could lead to suboptimal performance in terms of stability, robustness, and response time. Modern industrial applications, whose complexity and demands are constantly increasing, require more sophisticated and adaptive tuning methods that will allow real-time adjustments with better performance under changing operating conditions. New methodologies that will embody state-of-the-art optimization techniques are enabling considerable improvement in efficiency, accuracy, and reliability, hence guaranteeing optimal performance in various dynamic environments for the BLDC motor speed control.

**Limitation of existing techniques:**

Despite the pervasively applied traditional techniques and, more recently, metaheuristic algorithms for tuning controller gains in BLDC motor speed control, their shortcomings reveal the real need for new methodologies in this area. The currently developed metaheuristic algorithms are of a general problem of optimization and hence may not fully exploit the special dynamics of the BLDC motor, which results in nonoptimality in performance and high computational costs, which is quite impracticable for real-time applications. Traditional design methods of control, like the Ziegler-Nichols method, usually do not consider nonlinearities and complex dynamics, which results in manual tuning, a process that is inefficient and not adaptive.

The No Free Lunch (NFL) theorem is importance in optimization. The No Free Lunch Theorem states that there is no single optimization algorithm performing best on all problems, underlining the fact that it is precisely the generality of existing algorithms which conspires against their effectivity in specialized applications such as BLDC motor control. It encourages researchers in understanding the problem and helps them to develop or choose algorithms that are most suitable for the specific characteristics and requirements of the problem. This ultimately shows that new mechanisms are needed that provide improved adaptability, computational efficiency, and scalability, and are more robust to ensure the optimal performance in very different and dynamic environments. The NFL theorem proves to be a very important reminder of the intrinsic trade-offs in the design and selection of algorithms that enables more informed and effective decision-making in pursuit of the best solutions.

The following techniques applied in this work—Whale Optimization Algorithm, Particle Swarm Optimization, Accelerated PSO, variants of WOA, and Ant Colony Optimization; have their own special advantages in the tuning of PI controllers for speed control of BLDC motor. WOA achieves a very nice balance between exploration and exploitation, meant for passing through the complex, generally nonlinear parameter space of the PI controllers, with further adaptations for better adaptability to some specific motor dynamics that ensures robustness against different operational conditions. Incorporated Levy flight trajectory, the ability to take occasional large jumps prevent the algorithm to converge at local optima. Chaotic map provides a controlled randomization which plays a deterministic role in finding the optimal solution. PSO is usually a fast converging method to the optimal solution and is actually appropriate for real-time practices in which changes need to be fast. Its variants accommodate motor behavior dynamics, providing for improvement of control stability as well as response times. APSO has less computational time as it is a single update equation to proceed towards optimal solution. ACO has been proven robust against local optima, with a positive feedback mechanism that thereby guarantees reliable optimization in very large search spaces, as in PI controller tuning. These algorithms assure better performance in BLDC motor speed control, as they will enhance the system's accuracy, computational time, and adaptability in a better way as compared to the traditional methods.

**Significance of metaheuristic techniques:**

These algorithms have advantages over traditional tuning methods and other existing metaheuristic algorithms in some main aspects. As opposed to heuristic-based approaches that require manual adjustment and iterative testing, these techniques significantly reduce the time and effort needed for fine-tuning the PI controller gains by automating the optimization process. This ensures that optimal solutions are found more reliably and accurately, to improve control performance metrics such as stability, overshoot reduction, and response time. Unlike some metaheuristic algorithms, which might fall into highly dimensional or nonlinear optimization landscapes, variants of WOA, PSO, and ACO were designed to counteract these complexities and are therefore well-suited for the demanding dynamics of BLDC motors. It makes use of robust global search capabilities together with adaptive mechanisms to provide a more efficient and dependable PI controller tuning process for BLDC motor speed control systems in industrial applications.

We have incorporated the details of importance regarding the implemented meta-heuristic techniques in main manuscript. That paragraph is also mentioned below;

These metaheuristic techniques are preferable on other conventional techniques of tuning the PI controller's gains. Metaheuristic techniques follow an automated process of optimization which makes them different from other heuristic techniques. PSO technique depends on the population of searching agent and with iterative approach these agents move towards the best optimal solution. PSO is prefer because of its simplicity and fast rate of convergence. APSO offers an accelerated approach to converge at possible optimal solution as it has single update equation. ACO is another nature inspired technique which use the foraging behaviour and communication strategy of ants to traverse the whole search space by coordinating the following searcing particles to move towards the best-found solution. WOA offers a balance between exploration and exploitation which helps the search agents to give optimal solution within the search space and prevent premature convergence at any local optima. WOA is highly adaptive for complex and nonlinear problems. LFWOA incorporated Levy flight behaviour in the canonical version of WOA and enhance the exploration ability of the algorithm by taking random small steps with occasional long steps to avoid convergence at any local optima. Levy flight concepts is helpful in finding optimal solutions to complex and dynamic problems. Moreover, using chaotic map offers a controlled randomization which is helpful in finding optimal solution. The details of each algorithm are discussed in the following section.

4. The background of the research is rather weak. The authors should provide a more thorough overview of the scientific problem and show unresolved issues.

Response

We're grateful for your helpful input. Your idea to improve the manuscript's background section is highly appreciated.

We have involved more relevant as well as recent work of other researchers from literature to draw a clear picture of what basically is our main concern which is actually show how different algorithms are implemented and on just depending on instant value their supremacy over other is justified by showing their performance’s statistical comparison.

E. Celik 72 in 2024 proposed a new approach to tune cascade 1PDf-PI controller for BLDC motor using Snake Optimizer (SO). The study has shown better performance as compared to the traditional PI and 1PDf controllers, especially under varying speed reference and presence of external torque [19]. In 2022, Aoudni Y. did a study to enhance the resiliency of PID controllers in voltage and current control in such specialized domains of application. This study highlights the importance of advanced optimized techniques of controlling strategies for complex photonic and optical applications [20]. In 2022, Rodríguez-Molina et al. proposed a new approach to finely tune the speed controller if BLDC motor. In this approach a chaotic online differential evolution (CODE) algorithm to control the gains’ tuning of the controller in a chaotic manner. Results show that the CODE approach is very effective even in dynamic and uncertain conditions [21].

5. What is the need of a new algorithm, when effective optimization algorithms are available in the literature?

Response:

Thank you for your feedback. Even when many efficient optimization algorithms are available in the literature, there are a few reasons for which a new algorithm is needed. Although WOA, PSO, ACO, and many more techniques proved their efficiency in various optimization tasks, all of these techniques cannot be applied to specific problem domains like BLDC motor speed control. BLDC motors have special dynamic characteristics and operational constraints that cannot be captured by generic optimization algorithms. These motors also require precise adaptive control strategies that efficiently explore complex nonlinear parameter spaces and accommodate real-time adjustments to varying operational conditions. In this respect, a new algorithm designed specifically for the optimization of PI controller gains in BLDC motor speed control can exploit domain-specific insights and algorithms that result in improved accuracy, efficiency, and adaptiveness beyond that delivered by presently available methods. It will then ensure an effective process of optimization to better meet all the nuanced requirements of the BLDC motor control system, finally giving rise to improved performance and reliability in industrial applications.

6. Introduction is vaguely written. My suggestion is to divide the introduction into three subsections: 1) motivation and incitement, 2) literature review and 3) contribution and paper organization.

Response:

We would like to thank you for suggesting that the introduction should be divided into three subsections: 1) Motivation and Incitement, 2) Literature Review, 3) Contribution, and 4) Paper Organization. A good introduction serves to outline and situate research in such a way that its clarity on these aspects has to be very necessary. We have revised the introduction in light of your comment.

7. Improve the quality of figures.

Response:

Thank you for your constructive feedback regarding the quality of the figures presented in our manuscript. We appreciate this suggestion and realize that clear and quality visual displays are very important for properly enriching our research findings.

To enhance the quality of the figures, we have taken the following steps:

- Generated all figures with high resolution.
- Adjusted the colors and font size to make them clear and readable.
- Ensured that each figure has detailed and clear labels.

8. There is no discussion on the cost effectiveness of the methods surveyed. What is the computational complexity? Please include such discussions.

Response:

We regard your feedback highly. Cost-effectiveness and computational complexity issues are both vital for a full evaluation, and they need to be addressed in the surveyed optimization methods. We appreciate your valuable suggestion to include an elaborate computational complexity analysis for the algorithms in our study. While acknowledging this importance, as already indicated, our focus is comparative performance and efficacy of the algorithms for PI controller gain optimizations in BLDC motor speed control.

This study focuses on highlighting the practical benefits and performance improvements in control brought about by these optimization techniques. While computational complexity is an important measure, it falls slightly outside the primary scope of this research since we have focused more on proving the effectiveness and practical applicability of discussed algorithms, and their variants in achieving accurate adaptive control for BLDC motors. Any questions related to computational complexity or cost-effectiveness of the algorithms can be addressed in a rather large literature. In this way, we could still keep our study focused and clear but at the same time acknowledge the broader context of algorithm performance.

Thank you once again for your very valuable comment. We hope that with our explanation, the reasons for making such decisions in our scope of research are better understood.

9. There is no statistical test such as CEC 2019, 2017 and CEC 2022, to judge about the significance of the method's results. Without such a statistical test, the conclusion cannot be supported

Response:

Thank you for your highly valued comment on the kinds of statistical tests utilized to prove the results obtained from our method. We would really appreciate your suggestion in developing most of the tests, like CEC 2019, 2017, and CEC 2022, for inclusion.

We will highlight that, in this respect, very rigorous statistical tests have been performed, such as t-tests and Mann-Whitney U tests to probe the significance of our results. These are classical statistical techniques in the field of statistical analysis to compare means, differences between groups, and variance analysis. They provided very robust and meaningful insights both about the performance and significance of the proposed methods. While CEC tests in themselves forms a set of very important benchmarks within this field, we believe that our study's scope and objectives are adequately covered by the statistical tests utilized.

Thanks again for this constructive feedback.

**Response to Reviewer 2 Comments**

1. Introduction section should have a short summary at the end about the contents of the article for the readers. Kindly add.

Response:

First and foremost, we are grateful for your useful feedback on the structure of the introduction. We are also highly appreciative for pointing out that we should include a small summary at the end of the introduction to give readers an overview of what this article is about.

We have revised the introduction to include a concise summary that sketches out main sections and key points covered in the paper. This will be an addition to help guide readers through and clearly present an article map with respect to its structure and contributions.


2. Kindly present the previous works in a separate section, Literature review. This could be your section 2 as per traditional research methodology approach.

Response:

Thank you for your useful suggestion to provide a section dedicated to the literature review, and to present the previous works there. We have redefined the manuscript into a new section, Section 2, entitled "Literature Review." This will intend to give an overview of literature on research, and findings relevant to the optimization of PI controller gains for BLDC motor speed control. The revision is purposed at following the traditional approach of the methodology of research and introducing clarity and coherence into the paper.

3. Section 3 PI controller is more of a background, so it can be added as a subsection named Background in section 2, literature review.

Response:

Thank you for the kind suggestion about the structure of our manuscript. I have taken into consideration your proposal to include the PI controllers' section as a subsection under a "Background" heading within the literature review.

Most probably, the existing structure really suits our study. It is because we put the PI controller and its tuning methods at the core of our study, specifically on the investigation line of optimizing controller gains using metaheuristic algorithms.

I think if divide the introduction sector then it becomes more comprehend, rather than have a separate background section, with smooth transitions and flows from one piece of information to another.

4. Please redraw the Fig 2. Flow chart of implementation of metaheuristic algorithm-PI control BLDC motor, using suitable tool, it looks pretty wayward. Also, please mention YES/NO on every decision box to make it clear for readers. Use of colors is highly recommended for this flow chart.

Response:

Thank you for your feedback on Fig. 2: Flow Chart of the Implementation of Metaheuristic Algorithm - PI Control for BLDC Motor, to improve its clarity and readability. In view of your comments, we have revised the flow chart that would allow clearer structure and used colors to improve understanding. The decision boxes all have yes/no labels to firm up clear decision points for the readers, ensuring better comprehension of the algorithm workflow. You can view the attached main manuscript.

5. Kindly add fig. 2 in Methodology section instead of results section

Response:

Thank you for reaching out with your feedback about the placement of Fig. 2. We want to clarify that Fig. 2 is actually mentioned and cited in the Methodology section. However, because of some space and formatting constraints, it currently appears after the Results and Discussion section heading.

To make things clearer and ensure that the figure fits in the right place, we'll adjust the placement of Fig. 2 so that it appears directly within the Methodology section. This way, the figure will be more relevant to the context and easily accessible to readers right after the methodology description.

6. Kindly add simulation model specification in section 5 results and discussion instead of Methodology section.

Response:

Thank you for your feedback regarding the placement of the simulation model specification. We appreciate your suggestion to move this information to Section 5, Results and Discussion. In our opinion, it is more appropriate to keep the simulation model specification in the Methodology section. Including the simulation model specification here helps in maintaining a logical flow, where readers can first comprehend the methodological framework and then see how the results were derived based on that framework. It also allows for a more seamless transition from describing the methodology to presenting and interpreting the results.

We believe this structure enhances the clarity and coherence of the manuscript. However, we are open to further discussion if there are specific concerns or improvements that could be made regarding this placement.

7. It is strongly suggested to concise the section 5 in graphical form as it is convenient form for the readers to understand. The results are heavily populated with so many tables, try and replace as many with graphs.

Response:

Thank you so much for your valuable feedback. We totally get how important it is to present data in a clear and to-the-point way. While graphs and charts are great for getting a quick grasp of information, We think for Section 5, where we have some detailed results, it's best to use a table format. This is because all the data is super important and we need to present it in a comprehensive way to allow for a thorough analysis and interpretation. Tables give us a structured way to show precise values and comparisons, which is crucial for the level of detail in this study. Moreover, tabular representation is helpful for other researchers to compare their results in case they may reproduce the Simulink model and get comparable results.

8. Please include some relevant citations from year 2024. Also, it is recommended to cite more recent works from 2023 and 2022, since majority of references included are old.

Response:

Thanks for your insightful feedback! We totally get how important it is to include recent references to keep the research relevant and up to date. We'll dive into the literature and make sure to add more citations from 2023 and 2022, and even throw in a few from 2024. That way, our findings will have a more current context and solid support. This will help align the study with the latest advancements and discussions happening in the field.

**Response to Reviewer 3 Comments**

(1) The manuscript needs careful editing and particular attention to English grammar, spelling, punctuation marks and sentence structure.

Response:

Thank you so much for giving us your feedback on the language and writing quality of the manuscript. We really appreciate your attention to details and all your suggestions for improving grammar, spelling, punctuation, and sentence structure. We have actually used Grammarly to check the manuscript for any language issues, but we totally get that it is highly important to make sure the text is easy to read and sounds professional. So, we're going to do another thorough review of the manuscript, focusing specifically on grammar, spelling, punctuation, and sentence structure. We're even thinking about getting it reviewed by a professional editing service to make it even better.

(2) In the introduction, the references provided by the author are not up to date.

Response:

Thank you for your useful feedback. We now understand better how the study is supposed to have the latest references to make it pockets of relevance and accuracy. We revisited the introduction part to change and update with the latest references dating 2022, 2023, and 2024. This contextualizes the research in today’s time but puts it in tandem with the evolving concepts in the discipline.

(3) There are some typo errors in the manuscript, for instance, in Section 4, “trajectorybased” should be “trajectory based”.

Response:

Thank you for bringing out the typo errors in the manuscript. We have gone through the document and look for such errors and correct them. Notably, “trajectorybased” has been changed to “trajectory-based” in Section 4 of the manuscript. Your attention to detail is greatly acknowledged, and we ensured that there are no more typo mistakes in the final version of the manuscript.

(4) It is evident that there are some errors in the formulae, for instance, Eq. (1) and Eq. (2).

Response:

Thank you for pointing out errors in the formulae, especially in Eq. (1) and Eq. (2). We have looked over them carefully and correct these formulae to achieve their accuracy. Your feedback is very instrumental in enhancing the precision and reliability of the manuscript. For clarification we have also mentioned some detailed in text regarding these equations to abolish the confusion.

(6) The style of the images and tables is not uniform, and the quality is not optimal. It is recommended that the images and tables be modiﬁed to enhance their aesthetic appeal.

Response:

Thank you for your feedback regarding image and table style and quality. All tables and images will be uniform to achieve consistency in their appearance, creating a better look and feel of images and tables in terms of the quality of images, followed by consistency in formatting across all figures. Such suggestions shall be very useful in enhancing the readability and professional presentation of the manuscript as a whole.

(7) Some errors have been identiﬁed in the data entry process within the tables, for instance, Table 10.

Response:

Thank you for your feedback and observations regarding data entry on Table 10. We acknowledge your concern, and we want to clarify that results in the table were obtained using SPSS software. While going over the tables, we made sure numerical values are rounded up to an appropriate number of significant digits due to space limitations and for clarity and readability. This is typical formatting for style in scientific reporting so that all data are put in briefly yet accurately while reporting, within the space constraints of a manuscript.

It is much appreciated that you take great heed of minute details, and I shall do my best to present data clearly and precisely.

(8) It is imperative that the author elucidate the speciﬁc meanings of these indicators in the simulation.

Response:

Thank you for keen feedback. We value your suggestion to show what indicators used in the simulation entail. We have revised the manuscript to elaborate on the detailed explanations or definitions of each indicator stressing its meaning within the context of the simulation.

In case there are specific indicators wherein you found the phrasing not clear or requiring more explanation, please do bring it to my notice, and We will see that they are individually addressed in the revised manuscript itself. Your comments are imperative in making this simulation methodology and the results presented more lucid and understandable.
